# *ClBCM*: an EMS mutated gene regulating chlorophyll synthesis and adaptation to diverse stresses in watermelon

**DOI:** 10.1186/s43897-025-00223-6

**Published:** 2026-06-01

**Authors:** Taohong Fang, Zhen Zhuang, Mingxia Zhao, Xiao Chu, Jingsheng Tan, Xinyang Xu, Rui Cheng, Yi Zhang, Xu Wang, Yunbi Xu, Xingping Zhang, Yun Deng

**Affiliations:** 1 Shandong Provincial Key Laboratory of Precision Molecular Crop Design and Breeding, Peking University Institute of Advanced Agricultural Sciences, Shandong Laboratory of Advanced Agricultural Sciences in Weifang, Weifang, Shandong 261325 China; 2 Institute of Vegetables, Zhejiang Academy of Agricultural Sciences, Hangzhou, Zhejiang 310021 China; 3 Vegetable Research and Development Center, Huaiyin Institute of Agricultural Sciences of Xuhuai Region in Jiangsu, Huai’an, Jiangsu 223001 China

Watermelon (*Citrullus lanatus*) is a popular summer fruit cultivated worldwide, rich in nutrients such as sugars, lycopene, and citrulline. However, long-term domestication and selection have significantly reduced genetic diversity for important traits in cultivated watermelon, posing challenges for breeding stress-tolerant, high-quality, and productive varieties. Ethyl methane sulfonate (EMS) mutagenesis serves as a powerful approach to generate genetic variation for trait discovery (Deng et al. 2022; Zhang et al. 2024). Plant leaf is the main organ for photosynthesis and nutrient transformation. Chlorophyll (Chl) serves an important indicator for leaf color mutation (Wang and Grimm 2021). The genes underlying chlorophyll-deficient mutants remain largely unexplored in watermelon. Furthermore, most reported watermelon leaf color mutants are naturally occurring and non-stress-sensitive, such as the yellow leaf mutant *w-yl*, which exhibits yellow foliage throughout the entire growth period (Zhu et al. 2022), and the delayed green (*dg*) mutant, caused by a defect in the FtsH protease, which impairs chloroplast development in watermelons (Gebremeskel et al. 2023).

In this study, we characterized a stress-sensitive, stage-specific yellowing mutant (*g42yl*) derived from EMS mutagenesis of small-fruit watermelon inbreed G42 with reference genome (Deng et al. 2022). It displays G42-like green leaves at seedling (20–25 °C) and adult stages (28–40 °C), with deeper yellow flowers than the wild type. The leaves, stems and fruit skin of the mutant turn golden yellow at maturity (Fig. 1A; Fig. S1A). Under stresses including high temperature (HT), drought (DS), salt (SS), gummy stem blight (GSB), or mechanical damage stress (MS), *g42yl* shows leaf yellowing. However, the yellowing leaf color reverses to green upon stress removal (Fig. S1B, C; Fig. S2). This yellowing phenotype is primarily characterized by significant reductions of Chla (59.25%) and Chlb (73.82%), along with a decline in chlorophyll precursors (Proto IX, Mg-Proto IX, Pchlide) to 25–28% of G42 levels (Fig. 1B, C). Transmission Electron Microscope (TEM) revealed thylakoid reduction and grana degradation in *g42yl* chloroplasts under stress (Fig. 1D), indicating impaired chlorophyll metabolism and chloroplast development. Although the mutant has lower fruit weight and sugar content compared to the wild type (Fig. 1E, F), it remains a valuable genetic resource for breeding golden-skinned cultivars. Additionally, it is the first EMS-induced mutant that can act as an indicator of both plant stress status and fruit maturity.

**Fig. 1:**
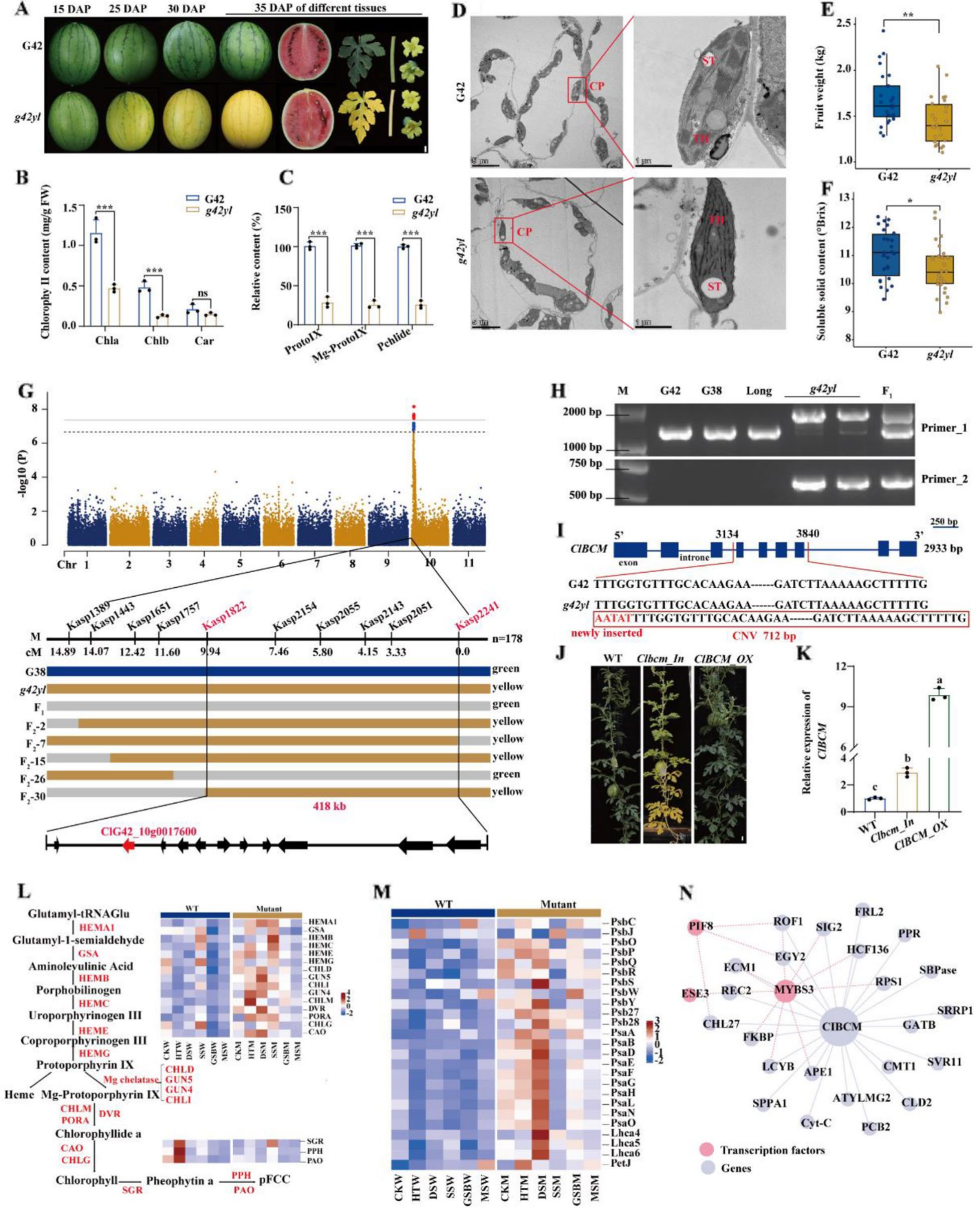
Characterization of the *g42yl* mutation in watermelon.** A** Phenotypes of mature organs in G42 and *g42yl*. DAP, days after pollination. **B** Chla, Chlb, and carotenoid levels in seedlings after 5 days of high temperature (HT). HT: day/night, 45℃/30℃. **C** Relative levels of chlorophyll precursor in *g42yl* vs. G42 under HT. Data: mean ± SD (*n* = 3). *** *P* < 0.001 (two-way ANOVA with Sidak’s test). **D** Chloroplast ultrastructure. CP, chloroplast; ST, starch granule; TH, thylakoid. **E–F** Fruit weight (**E**) and soluble solid content (**F**). Boxes: the IQR (Interquartile Range); Central line: median. *P < 0.05, **P < 0.01 (Unpaired t test). **G** Fine mapping of yellowing gene *ClBCM* on watermelon. **H** Detection of copy number variation (CNV) in ClG42_10g0017600 by electrophoresis. Amplification of the whole insertion (Primer_1) and the CNV breakpoint (Primer_2). **I** The gDNA structure of *ClBCM*. **J** WT, *Clbcm_In* and *ClBCM_OX* phenotypes. Scale bar = 2 cm. **K** Relative expression of *ClBCM*. Data: mean ± SD (*n* = 3). Different letters: significant differences (*P* < 0.05, Tukey’s HSD test). **L-M** Chlorophyll metabolism (**L**) and photosystem (**M**) differentially expressed genes (DEGs). **L** (Left) chlorophyll metabolism pathways (enzymes: red, products: black). (Right) Expression profile of biosynthetic (upper) and degradative (lower) enzyme genes in WT and mutant under stress. Data: log_2_(TPM + 1) (Transcripts Per Million) transformed and row-scaled. CK, control; DS, drought stress; SS, salt stress (250 mM NaCl, bottom irrigation); GBS, gummy stem blight; MS, mechanical stress. HEMA1, glutamyl-tRNA reductase; GSA, Glutamate 1-semialdehyde aminotransferase; HEMB, porphobilinogen synthase; HEMC, porphobilinogen deaminase; HEME, uroporphyrinogen decarboxylase; HEMG, protoporphyrinogen oxidase; CHLD, magnesium chelatase D subunit; GUN4/5, GENOMES UNCOUPLED 4/5; CHLI, magnesium chelatase I subunit; CHLM, magnesium proto IX methyltransferase; DVR, 8-Vinyl reductase; PORA, NADPH protochlorophyllide oxidoreductase; CHLG, chlorophyll synthase; CAO, chlorophyllide an oxygenase. SGR, STAY-GREEN; PPH, pheophytinase; PAO, pheophorbide a oxygenase. **N** Network of *ClBCM*-associated genes and transcription factors (TFs). Gray solid: weighted gene co-expression network analysis (WGCNA) connections (weight > 0.2). Red dashed: predicted TF regulation based on Finding Informative Motif Occurrences (FIMO)

We identified the mutant trait as being controlled by a single recessive nuclear gene, based on the segregation ratio of green and yellow individuals in the *g42yl*/G38 F_2_ population (Table S1). The identified candidate gene, *ClBCM* (ClG42_10g0017600), encodes a chloroplast-localized CAAX protease self-immunity protein with 370 amino acids (Fig. 1G; Fig. S3; Table S2-S5). The mutant phenotype is caused by a 712-bp insertion at the last 6 bp of the seventh exon of *ClBCM*, where the first 5 bp was the newly inserted base AATAT and the last 707 bp was a repeat of the original fragment (the repetition range was from 56 bp before the fourth exon to 6 bp after the seventh exon), named *Clbcm_In* (Fig. 1H, I). Meanwhile, Dr. Yang at Henan Agricultural University identified a 90-bp deletion in *ClBCM* in the natural yellow mutant WM103, named *Clbcm_Del* (personal communication). We compared the two mutants under different stresses and verified that they displayed identical phenotype (Fig. S1B). The *ClBCM_*over-expression in *g42yl* showed green leaf color and green fruit skin under high-temperature condition (Fig. 1J, K; Fig. S4). *ClBCM* is upregulated by aging and stresses. Its expression gradually increased during seedling development (21–42 days) and under high temperature stress (0–7 days) (Fig. S5A). In the mutant, *Clbcm_In* and chlorophyll degradation/senescence-related genes (SGR, PPH, ORE1) exhibited rapid upregulation during early stress exposure, which may account for the faster chlorophyll degradation observed in the mutant leaves (Fig. 1P; Fig. S5B-G). Lhcb6 (CP24) and Lhcb4 (CP29) are the PSII components, and it has recently been reported that BCM1 prevents CP24 and CP29 from degradation under high temperature stress to confer resistance to adversity (Li et al. 2025). A dramatic decline in CP24 and CP29 expression during the early stages of stress confirms that these proteins undergo accelerated degradation without *ClBCM*-mediated protection (Fig. S5H, I). These results indicate that *ClBCM* is activated during aging or under stress, promoting chlorophyll synthesis and participating in the regulation of stress responses. Disruption of *ClBCM* function impairs chlorophyll supply in mature or stressed leaves, leading to premature senescence and yellow appearance.

To explore mechanisms underlying leaf yellowing, we compared transcriptomes of G42 and *g42yl* under control (CK) and five stresses (HT, DS, SS, GSB, MS) (Fig. S6A-C; Table S6-S8). Both under control and stress treatments, the mutant displayed elevated transcript levels of chlorophyll biosynthesis and photosystem genes than the WT (Fig. 1L, M; Table S9), demonstrating that the *ClBCM* mutation reshapes the expression of chloroplast-associated genes to compensate for its physiological impact. However, the mutant displayed greater stress sensitivity with leaf chlorosis, suggesting that disrupted chloroplast gene regulation may trigger premature senescence. WGCNA analysis identified 23 *ClBCM*-associated genes and three TFs (PIF8/ESE3/MYB3) (Fig. 1N; Fig. S6D, E; Table S10-S12). Among these, EGY2 has been previously reported to interact with *BCM1* and participate in chlorophyll synthesis (Fu et al. 2025). The FKBP (FK506-binding protein) family member ROF1 (FKBP62) and RPS1 (Ribosomal Protein S1) have been reported to be involved in plant responses to high-temperature stress (Meiri and Breiman 2009; Yu et al. 2012). PPR proteins participate in chloroplast RNA splicing and are involved in plant development and stress responses (Tan et al. 2014). Additionally, we predicted that the transcription factors PIF8, ESE3, and MYBS3 have binding sites in the genes ROF1, RPS1, and EGY2. However, whether these genes and transcription factors directly or indirectly interact with *ClBCM* to participate in stress responses remains to be further verified. The sensitivity of chloroplasts to stress can trigger adaptive changes in nuclear gene expression, but the role of *ClBCM* in the signaling from chloroplasts to the nucleus remains to be explored. Tetrapyrroles are central to retrograde signaling, with Mg-Proto IX plays a key role in this signaling cascade (Barajas-López et al. 2013). *ClBCM* is known to cooperate with EGY1 and GUN4 to stimulate Mg-chelatase activity, converting Proto IX to Mg-Proto IX (Fu et al. 2025; Wang et al. 2020). Under stress conditions, the *ClBCM* mutation caused a reduction in Mg-proto IX levels to below 50%, which may weaken the signal transmitted from chloroplasts to the nucleus, preventing the plant from rapidly responding to stress and activating defense mechanisms.

Collectively, our study demonstrates that *ClBCM* serves as a crucial stress-responsive gene that promotes chlorophyll biosynthesis, responding not only to heat stress but also to drought, salinity, and pathogen challenges. We propose that expression of this gene is stress-induced and regulates pigment metabolism pathways, with signals like Mg-proto IX transmitted to the nucleus to activate expression of nuclear-encoded plastid-related genes for stress response. We identified several novel genes potentially involved in regulating BCM-mediated stress responses, including APE1, RPS1, FKBP, and HCF136, as well as the transcription factors PIF8, ESE3, and MYBS3, all of which require further validation. Elucidating the underlying mechanisms will provide valuable genetic bases for developing stress-resistant watermelon varieties and golden-rind cultivars via molecular breeding. In addition, this yellow mutation can serve as a useful indicator for whether the plants suffer from stresses or fruits are matured.

## Supplementary Information

Supplementary Material 1.

Supplementary Material 2.

Supplementary Material 3.

## Data Availability

Data are available upon request to the corresponding author.

## References

[CR1] Barajas-López JD, Blanco NE, Strand Å. Plastid-to-nucleus communication, signals controlling the running of the plant cell. Biochim Biophys Acta. 2013;1833(2):425–37. 22749883 10.1016/j.bbamcr.2012.06.020

[CR2] Deng Y, Liu S, Zhang Y, Tan J, Li X, Chu X, et al. A telomere-to-telomere gap-free reference genome of watermelon and its mutation library provide important resources for gene discovery and breeding. Mol Plant. 2022;15(8):1268–84. 35746868 10.1016/j.molp.2022.06.010

[CR3] Fu D, Zhou H, Grimm B, Wang P. The BCM1-EGY1 module balances chlorophyll biosynthesis and breakdown to confer chlorophyll homeostasis in land plants. Mol Plant. 2025;18(1):76–94. 39628053 10.1016/j.molp.2024.11.016

[CR4] Gebremeskel H, Umer MJ, Hongju Z, Li B, Shengjie Z, Yuan P, et al. Genetic mapping and molecular characterization of the delayed green gene *dg* in watermelon (*Citrullus lanatus*). Front Plant Sci. 2023;14:1152644. 37152178 10.3389/fpls.2023.1152644 PMC10158938

[CR5] Li Q, An W, Ma J, Zhang H, Luo M, Qi Y, et al. The thylakoid protein BCM1 sequesters antennae protein CP24 and CP29 within the grana cores thereby reducing their exposure to degradation under heat stress. Plant J. 2025;121(5):e70060. 40026239 10.1111/tpj.70060

[CR6] Meiri D, Breiman A. *Arabidopsis* ROF1 (FKBP62) modulates thermotolerance by interacting with HSP90.1 and affecting the accumulation of HsfA2-regulated sHSPs. Plant J. 2009;59(3):387–99. 19366428 10.1111/j.1365-313X.2009.03878.x

[CR7] Tan J, Tan Z, Wu F, Sheng P, Heng Y, Wang X, et al. A novel chloroplast-localized pentatricopeptide repeat protein involved in splicing affects chloroplast development and abiotic stress response in rice. Mol Plant. 2014;7(8):1329–49. 24821718 10.1093/mp/ssu054

[CR8] Wang P, Grimm B. Connecting chlorophyll metabolism with accumulation of the photosynthetic apparatus. Trends Plant Sci. 2021;26(5):484–95. 33422426 10.1016/j.tplants.2020.12.005

[CR9] Wang P, Richter AS, Kleeberg JRW, Geimer S, Grimm B. Post-translational coordination of chlorophyll biosynthesis and breakdown by BCMs maintains chlorophyll homeostasis during leaf development. Nat Commun. 2020;11(1):1254. 32198392 10.1038/s41467-020-14992-9 PMC7083845

[CR10] Yu HD, Yang XF, Chen ST, Wang YT, Li JK, Shen Q, et al. Downregulation of chloroplast RPS1 negatively modulates nuclear heat-responsive expression of HsfA2 and its target genes in Arabidopsis. PLoS Genet. 2012;8(5):e1002669. 22570631 10.1371/journal.pgen.1002669 PMC3342936

[CR11] Zhang Y, Zhao M, Tan J, Huang M, Chu X, Li Y, et al. Telomere-to-telomere *Citrullus* super-pangenome provides direction for watermelon breeding. Nat Genet. 2024;56(8):1750–61. 38977857 10.1038/s41588-024-01823-6 PMC11319210

[CR12] Zhu Y, Yuan G, Wang Y, An G, Li W, Liu J, et al. Mapping and functional verification of leaf yellowing genes in watermelon during whole growth period. Front Plant Sci. 2022;13:1049114. 36340411 10.3389/fpls.2022.1049114 PMC9627507

