# Supplementary material for: ClBCM: an EMS mutated gene regulating chlorophyll synthesis and adaptation to diverse stresses in watermelon
Source: Mol Hortic. 2026 Jun 1;6:39. doi: 10.1186/s43897-025-00223-6 (PMC13224701; doi:10.1186/s43897-025-00223-6)
Supplement: Supplementary file 1 — Supplementary Material 1. [file 43897_2025_223_MOESM1_ESM.docx]

**Source data for Fig. 1H**


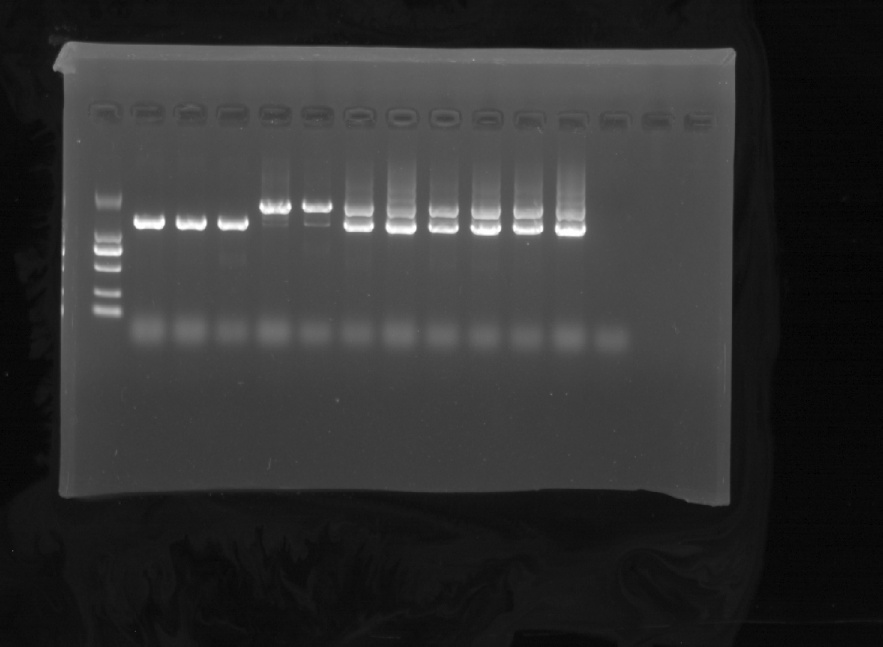


2000 bp

1000 bp

M

G42

G38

Long

*g42yl*

G42/*g42yl* F1

G38/*g42yl* F1

Long/*g42yl* F1

*g42yl*/G42 F1

*g42yl*/G38 F1

*g42yl*/Long F1

H2O

1367 bp

2076 bp


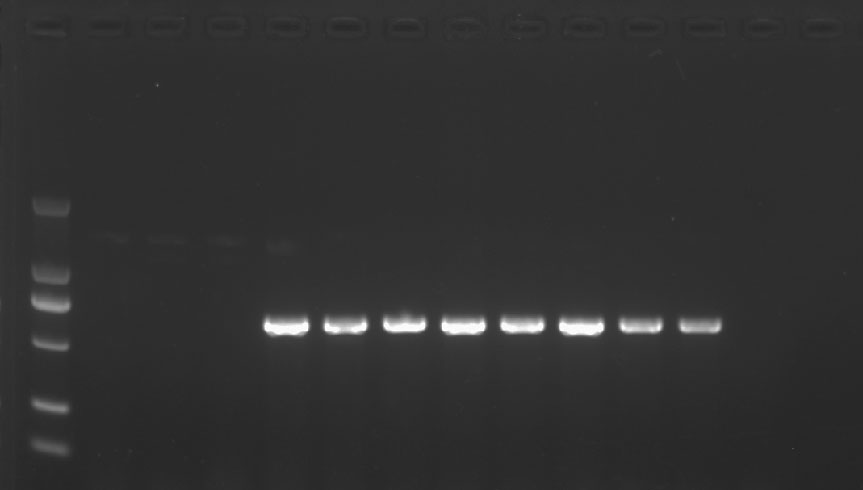


750 bp

1000 bp

M

G42

G38

Long

*g42yl*

G42/*g42yl* F1

G38/*g42yl* F1

Long/*g42yl* F1

*g42yl*/G42 F1

*g42yl*/G38 F1

*g42yl*/Long F1

H2O

500 bp

644 bp

**Source data for Fig. S4C**


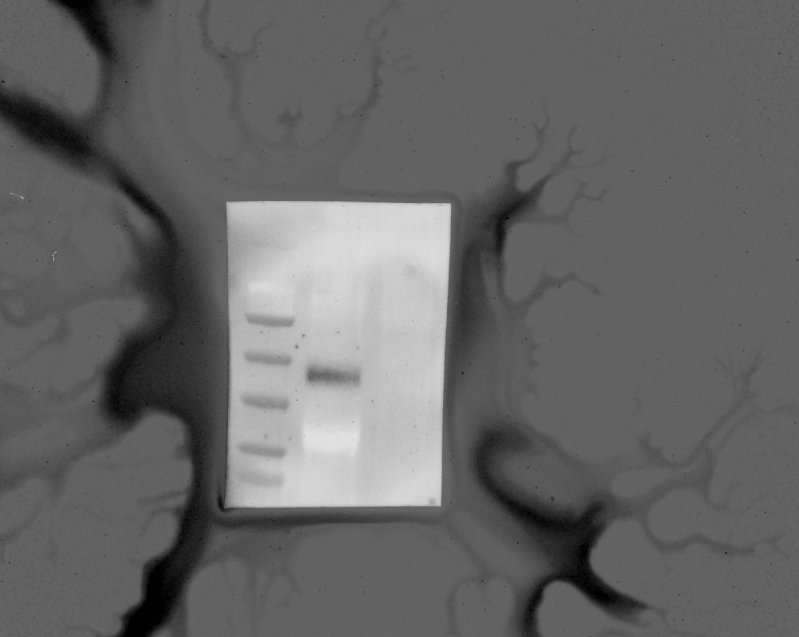


43

55

33

26

kDa

ClBCM_OX

Clbcm_In
