# Supplementary material for: ClBCM: an EMS mutated gene regulating chlorophyll synthesis and adaptation to diverse stresses in watermelon
Source: Mol Hortic. 2026 Jun 1;6:39. doi: 10.1186/s43897-025-00223-6 (PMC13224701; doi:10.1186/s43897-025-00223-6)
Supplement: Supplementary file 2 — Supplementary Material 2. [file 43897_2025_223_MOESM2_ESM.docx]

**Materials and methods**

**Plant materials, growth conditions, and phenotypic data collection**

The leaf color yellowing mutant (*g42yl*) was derived from a EMS-treated population of small-fruited diploid watermelon inbred line G42. In this study, the mutant was crossed with G42, and G38, respectively and the resulting F_1_ plants were self-pollinated to generate two F_2_ populations. Parents and populations were planted at the Experimental Station of the Peking University Institute of Advanced Agricultural Sciences, Weifang, Shandong in multi-span plastic greenhouse in 2022, 2023, and 2024. Prior to stress treatment, both wild-type and mutant were grown for three weeks on soil in a plant growth chamber (FH740) under standard conditions (16/8 h photoperiod, light intensity 30,000 Lx) and a 25 °C/20 °C (day/night) temperature cycle at relative humidity of 60%. Different stress conditions include: high temperature (day/night, 45℃/30℃), drought stress (plants were subjected to continuous drought until leaf wilting occurred), salt stress (250 mM NaCl, bottom irrigation.), gummy stem blight (GSB), and low temperature (day/night, 10℃/4℃), with standard conditions as control. Pathogen inoculation: apply 5 drops (10 µL/drop) of spore suspension (1×10^-6^ spores/mL) to leaf axils and surfaces on two leaves per plant. Incubate in darkness with high humidity for 48 hours before transferring to normal growth conditions for disease development. Each stress treatment consisted of three biological replicates, each comprising 50 seedlings. After being subjected to stress for 5 days, plants were sampled for phenotypic observation and chlorophyll analysis.

**Determination of pigment content and chlorophyll precursor**

Fresh leaf samples were collected from the second true leaves of 21-day-old seedlings, with each sample comprising leaves from five plants per genotype and three biological replications. We measured chlorophyll content in overexpression plants by sampling leaves from the fruit-bearing nodes after a 5-day high-temperature stress treatment at the fruit-setting stage.

For chlorophyll and carotenoid quantification, 0.05 g samples were homogenized in 5 mL 80% acetone using 15 mL conical tubes, and 24 h dark incubation at room temperature until the leaves turned completely white. The absorbance A663, A645 and A470 at 663 nm, 645 nm and 470 nm were determined by UV spectrophotometer (UV-2600I, Shimadzu, Kyoto, Japan). The concentrations of chlorophyll a (Chla), chlorophyll b (Chlb), and carotenoids (Car) were calculated using 80% acetone as control. The equations are as following (Lichtenthaler *et al*., 1987):

Chla(mg/g FW)=(12.7×A663-2.69×A645)×V/W

Chlb(mg/g FW)=(22.9×A645-4.68×A663)×V/W

Car(mg/g FW)=(1000×A470×V/W-3.27×Chla-104×Chlb)/198.

where V represents the total volume of extract (L), and W represents leaf mass (g).

The contents of main chlorophyll precursor in the process of chlorophyll synthesis were measured. Fresh leaves (0.5 g) were collected and grounded in 5 mL extraction buffer (80% acetone), diluted to 10 mL, and then centrifuged at 13,000 g for 10 min. The absorbance values of the supernatants were measured at 575, 590 and 628 nm, respectively. The contents of protoporphyrin IX (Proto IX, Mg-protoporphyrin IX (Mg-proto IX), and protochlorophyllide (Pchlide) were calculated by the following equations (Hodgins *et al.*, 1986):

Mg-ProtoIX(ug/g FW)=0.06077×A590-0.01937×A575-0.003423×A628

ProtoIX(ug/g FW)=0.18016×A575-0.04036×A628-0.04515×A590

Pchlide(ug/g FW)=0.03563×A628+0.007225×A590-0.02955×A575

**Ultrastructural observation of chloroplast**

The 20th true leaves with obvious color differences from G42 and *g42yl* at the fruit ripening stage were collected for transmission electron microscopy (TEM) observation. These leaves were cut into small pieces of 0.1 mm × 0.2 mm size and fixed in 4.0% glutaraldehyde solution and 1% osmium tetroxide. Leaf samples were prepared for TEM according to Zhu *et al*. (2022).

**Mapping and identification of the leaf yellowing gene**

Two F_2_ populations (*g42yl*/G42 and *g42yl*/G38) were constructed to elucidate the inheritance pattern of the chlorosis-controlling gene. Since we found that G38 has a more diverse genetic background compared to *g42yl*, with higher genetic polymorphism, enhanced mapping accuracy and amplified phenotypic variation, the *g42yl*/G38 F_2_ population was utilized for mapping. Leaf DNA of 30 individual plants with yellowed and green extreme phenotypes in the *g42yl*/G38 F_2_ population were selected for the whole-genome sequencing, and parental DNA was used to construct the parental pools for sequencing analysis. The sequencing libraries were constructed according to the manufacturer’s instructions (MGI Tech Co., Ltd., Shenzheng, China). Sequencing was performed by Kindstar Sequenon Co., Ltd. (Hubei, China) using DNBSEQ-T7 PE150 and the average sequencing depth was 30×. The raw paired-end reads were trimmed to remove adaptors and low-quality bases using Fastp (v.0.23.4). The reads were filtered with a sliding window of size 5, with an average Phred score of 20 within the window, and reads containing more than 5 N bases were also removed. All clean reads for each accession were aligned to the G42 (http://www.watermelondb.cn/#/jbrowse?version=G42) genome using the ‘MEM’ algorithm in the Burrows-Wheeler Aligner (bwa-mem v0.7.17-r1188) (Li *et al.*, 2009). After sorting the reads using Samtools (v.1.9) (Danecek *et al.*, 2021), redundant reads were removed using Picard(v.2.18.29) (<http://broadinstitute.github.io/picard/>). The HaplotypeCaller module in GATK (v.4.4.0.5.1) (McKenna *et al.*, 2010) was used to generate gvcf files for each accession and then to identify SNPs and InDels in the panel. SNP and InDel annotations were conducted based on the G42 genome using ANNOVAR (v.2020-06-07) (Wang *et al.*, 2010). The original SNPs were further filtered following the criterion that only SNPs or InDels with a minor allele frequency greater than 5% and less than 20% missing data were considered high-quality ones. Finally, a total of 162,243 high-quality SNPs and 69,765 high-quality InDels were obtained and used for further analysis.

GWAS for the yellowing was performed using the SNP and InDel data under the MLM (Mixed Linear Model) using the TASSEL (v.5.2.89) (Wang *et al.*, 2007) software package. A kinship (K) matrix in the Centered IBS method of TASSEL was used to correct the population structure. The significance threshold of SNP-INDEL-trait associations was established with a false-detection-rate-adjusted *P* < 0.05 using the Benjamini-Hochberg procedure (Benjamini *et al.*, 1995), which corresponds to an uncorrected *P* value of approximately 2.174083e-07.

**Data statistical analysis and genetic linkage mapping**

All data were organized using Microsoft Office 2021, and graphs were generated along with statistical analyses performed using GraphPad Prism 8 and Adobe Illustrator. Chi-square two-tailed test was used to assess whether the F₂ populations conformed to a 3:1 ratio. Unpaired two-tailed Student’s t-test was applied to determine significant differences in fruit weight, central sugar, chlorophyll and its precursor levels between wild-type and mutant plants. Statistical significance was determined using the Holm-Sidak method, with alpha = 0.05. Significance asterisks are: ns (*P*>0.05), * (*P* < 0.05), ** (*P* < 0.01), *** (*P* < 0.001).

The genetic distance among KASP markers were calculated using QTL IciMapping V4.2 software, and Kosambi function was used to calculate the map distance (cM) in centimorgans. The likelihood odds of LOD (LOD) were calculated by the software arrangement test, with the threshold set to 2.5 and the permutation value set to 1000 times (*P*=0.01). Use the IBS software (https://www.ibs.renlab.org/#/home) to draw the gene structure diagram. Use the online platforms: https://www.bioinformatics.com.cn/ and <https://www.omicshare.com/tools/Home/Soft/getsoft> to plot transcriptome heatmaps.

**RNA sequencing and weighted gene co-expression network analysis**

To investigate the gene expression profiles that affect leaf yellowing, transcriptome sequencing of G42 and *g42yl* across six treatments including control, high temperature, drought stress, salt stress, gummy stem blight and mechanical stress were performed. The first-true leaves of three plants were selected as a sample from G42 and *g42yl*, each with three biological replications. The cDNA library was sequenced by Illumina Nova xplus system (Novogene Co., Ltd. -Beijing, China) with reads of 150bp in length. The raw paired-end reads were trimmed to remove adaptors and low-quality bases using Fastp ( v.0.23.4)，The reads were filtered with a sliding window of size 5, with an average Phred score of 20 within the window, and reads containing more than 5 N bases were also removed. Then clean reads were mapped into the ‘G42’ watermelon genome (http://www.watermelondb.cn/#/jbrowse?version=G42) using HISAT2 (v2-2.2.1). BAM files were used to calculate the expression level of each gene by StringTie (v2.2.1) (Pertea *et al.*, 2015). Differentially expressed genes (DEGs) were identified using DEseq2 package (v.1.38.3) with gene fold change ≥2.00 and false discovery rate ≤0.05. The gene expression levels were based on the numbers of Transcripts Per Million (TPM). Enrichment analyses of Gene Ontology (GO) and Kyoto Encyclopedia of Genes and Genomes (KEGG) for DEGs were performed using cluster Profiler package (v.4.6.2) (Yu *et al.*, 2012) with the cutoff of *p* < 0.05. The R package WGCNA (v.1.72.5) (Langfelder et al., 2008) was used to construct a co-expression network and to identify the modules of highly correlated genes based on the normalized expression matrix of DEGs. The modules were obtained using the automatic network construction function blockwise with default settings, except that the soft power was set to 16, the minimal module size was set to 50, and the merge cut height was set to 0.25. The binding potential between transcription factors and the promoter regions of target genes was predicted using FIMO (v5.5.1) (Grant *et al.*, 2011).

**QPCR validation and gene expression analysis**

To characterize *ClBCM* expression dynamics, the first-true leaf from 21-day-old (two-true leaf) wild-type plants were sampled at 7-day intervals under standard growth conditions (25°C/20℃, 16 h light/8 h dark, 60% RH) until 42d. For stress response analysis, we subjected the plants to high-temperature treatment (45°C day / 30°C night) when they grew to two true leaves, and took the leaves on 0, 3, 5, and 7 days after treatment. Three biological replicates of 0.1 g leaf samples were collected at indicated time points from the first true leaf. Additionally, 0.3 g leaves were harvested from the second flower node (fruit-setting stage) of WT, mutant, and transgenic plants, also with three biological replicates per genotype. Leaf RNA was extracted using the FastPure Universal Plant Total RNA Isolation Kit (#RC411-01, Vazyme, Nanjing, China). Total RNA samples of 1 μg were then reverse transcribed into cDNA using the HiScript III All-in-one RT SuperMix Perfect for qPCR (#R333-01, Vazyme). Quantitative RT-PCR assays were performed using the ChamQ SYBR qPCR Master Mix (#Q711-02, Vazyme). The actin (Cla97C02G026960/ClG42_02g0007100) gene of watermelon was used as the internal reference to calibrate the expression level of the gene to be analyzed (Wang *et al.*, 2021). The reference gene 2-ΔΔCt method was employed to quantify relative gene expression levels and normalized on the basis of the expression level of candidate genes (Livak *et al.*, 2001). The PCR primers used are listed in Table S13.

**Transgene constructs and subcellular localization of *ClBCM***

For overexpress *ClBCM* in the mutant and observation of subcellular localization signals of the *Clbcm_In* and *ClBCM*, the 1113-bp CDS sequence of *ClBCM* from the G42 and 1482-bp CDS sequence of *Clbcm_In* from the *g42yl* were fused to the N-terminal of the green fluorescent protein gene (*GFP*) under the control of the *35S* promoter in the pZHA702-pZKD662-PTG vector, respectively. The primer sequences for construction of these vectors are listed in Supplementary Table S13. Agrobacterium tumefaciens strain GV3101 carrying recombinant vector was cultured on YEP (Yeast Extract Peptone Broth Medium) with appropriate antibiotics at 28℃ for 12-16h, resuspended in infiltration buffer [10 Mm MgCl_2_, 10 Mm 2-(N-morpholino) ethanesulfonic acid (MES), pH 5.6, 150 µm acetosyringone] at an optical density at 600 nm (OD600) of 0.6 or 0.8, and infiltrated into expanded leaves of 4-week-old *N. benthamiana*. After 2 days post-infiltration, the subcellular localization of Fluorescence was visualized and photographed using excitation/emission filters for EGFP fluorescence (Ex/Em, 486/480-550 nm) and Chl fluorescence (Ex/Em, 562/570-718 nm) with a confocal laser-scanning microscope Leica Nikon A1R (Leica Microsystems, Wetzlar, Germany).

**Protein extraction and western-blot analysis**

To extract thylakoid membrane proteins from heat-treated transgenic mature leaves, 4 g of leaf tissue from the second flower node (fruit-setting stage) of transgenic plants were homogenized in approximately 20 mL of HMSN buffer (10 mM HEPES/KOH, pH 7.6, 5 mM MgCl_2_ ·6H_2_O, 0.4 mM sucrose and 10 mM NaCl) using a pre-chilled mortar and pestle, followed by filtration into a centrifuge tube. The homogenate was centrifuged at 3,000 × g for 10 min at 4°C, and the supernatant was discarded to obtain the crude thylakoid membrane fraction. Add 1 mL of PEB buffer (2% [w/v] SDS, 56 mM Na₂CO₃, 12% [w/v] sucrose, and 2 mM EDTA, pH 8.0) to 100 μL of crude thylakoid membranes. The BCM protein extraction method was performed as previously described by Wang *et al.* (2020). After electrophoresis, the proteins were transferred to 0.45 μM PVDF membrane (Merck Millipore, Germany) and probed with an anti-FLAG antibody at a dilution of 1:10,000.

**References**

Lichtenthaler HK. Chlorophylls and carotenoids: pigments of photosynthetic biomembranes. Methods in Enzymol. 1987; 148:350-82.

Hodgins RR, Huystee RB. Rapid simultaneous estimation of protoporphyrin and Mg-protophyrins in higher plants. J Plant Physiol. 1986;1 25:311-23.

Li H, Durbin R. Fast and accurate short read alignment with Burrows-Wheeler transform. Bioinformatics. 2009; 25(14):1754-60.

Danecek P, Bonfield JK, Liddle J, Marshall J, Ohan V, Pollard MO, Whitwham A, Keane T, McCarthy SA, Davies RM, Li H. Twelve years of SAMtools and BCFtools. Gigascience. 2021; 10(2):giab008.

McKenna A, Hanna M, Banks E, Sivachenko A, Cibulskis K, Kernytsky A, Garimella K, Altshuler D, Gabriel S, Daly M, DePristo MA. The Genome Analysis Toolkit: a MapReduce framework for analyzing next-generation DNA sequencing data. Genome Res. 2010; 20(9):1297-303.

Wang K, Li M, Hakonarson H. ANNOVAR: functional annotation of genetic variants from high-throughput sequencing data. Nucleic Acids Res. 2010; 38(16):e164.

Bradbury PJ, Zhang Z, Kroon DE, Casstevens TM, Ramdoss Y, Buckler ES. TASSEL: software for association mapping of complex traits in diverse samples. Bioinformatics. 2007; 23(19):2633-5.

Benjamini Y, Hochberg Y. Controlling the false discovery rate: a practical and powerful approach to multiple testing. J R Stat Soc Ser B*.* 1995; 57:289-300.

Pertea M, Pertea GM, Antonescu CM, Chang TC, Mendell JT, Salzberg SL. StringTie enables improved reconstruction of a transcriptome from RNA-seq reads. Nat Biotechnol. 2015; 33(3):290-5.

Yu G, Wang LG, Han Y, He QY. clusterProfiler: an R package for comparing biological themes among gene clusters. OMICS. 2012; 16(5):284-7.

Langfelder P, Horvath S. WGCNA: an R package for weighted correlation network analysis. BMC Bioinformatics. 2008; 9:559.

Grant CE, Bailey TL, Noble WS. FIMO: scanning for occurrences of a given motif. Bioinformatics. 2011; 27(7):1017-8.

Wang J, Wang Y, Zhang J, Ren Y, Li M, Tian S, Yu Y, Zuo Y, Gong G, Zhang H, Guo S, Xu Y. The NAC transcription factor ClNAC68 positively regulates sugar content and seed development in watermelon by repressing ClINV and ClGH3.6. Hortic Res. 2021; 8(1):214.

Livak KJ, Schmittgen TD. Analysis of relative gene expression data using real time quantitative PCR and the 2-ΔΔCt method. Methods. 2001; 25:402-8.

**Supplementary figures**


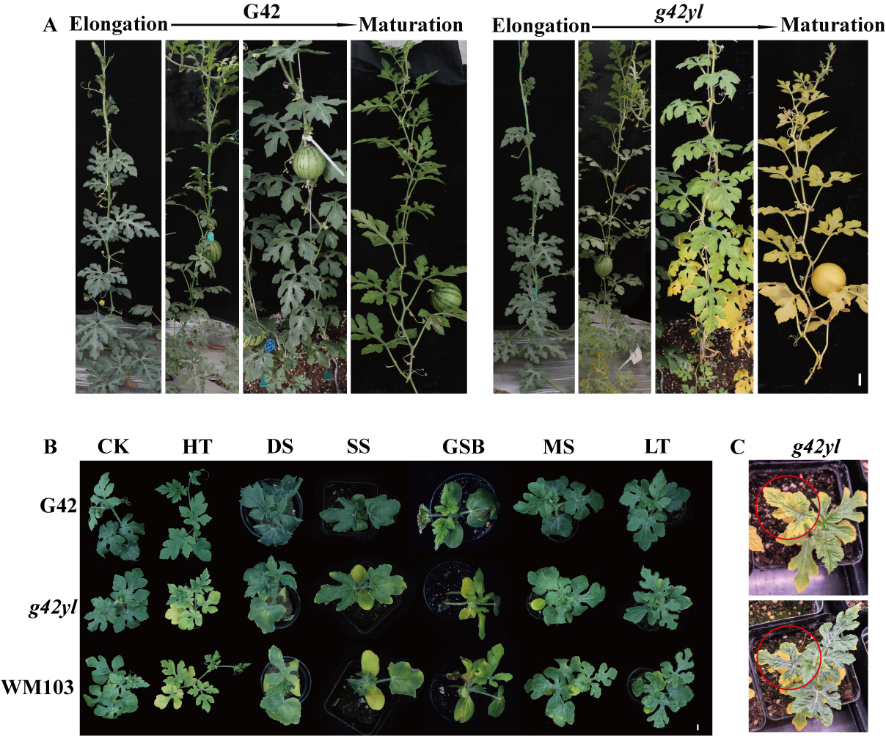


**Fig. S1 Plant phenotypes of G42, *g42yl* and WM103.** (A) Plant phenotypes of G42 and *g42yl* at adult stages. (B) Seedling phenotypes after 5 days of various stress treatments. CK, control; HT, high temperature (day/night, 45℃/30℃); DS, drought stress; SS, salt stress (250 mM NaCl, bottom irrigation); GBS, gummy stem blight; MS, mechanical stress; LT, low temperature (day/night, 10℃/4℃). Scale bar=1cm. (C) Color changes in mutant seedlings from HT (upper) to recovery (lower).


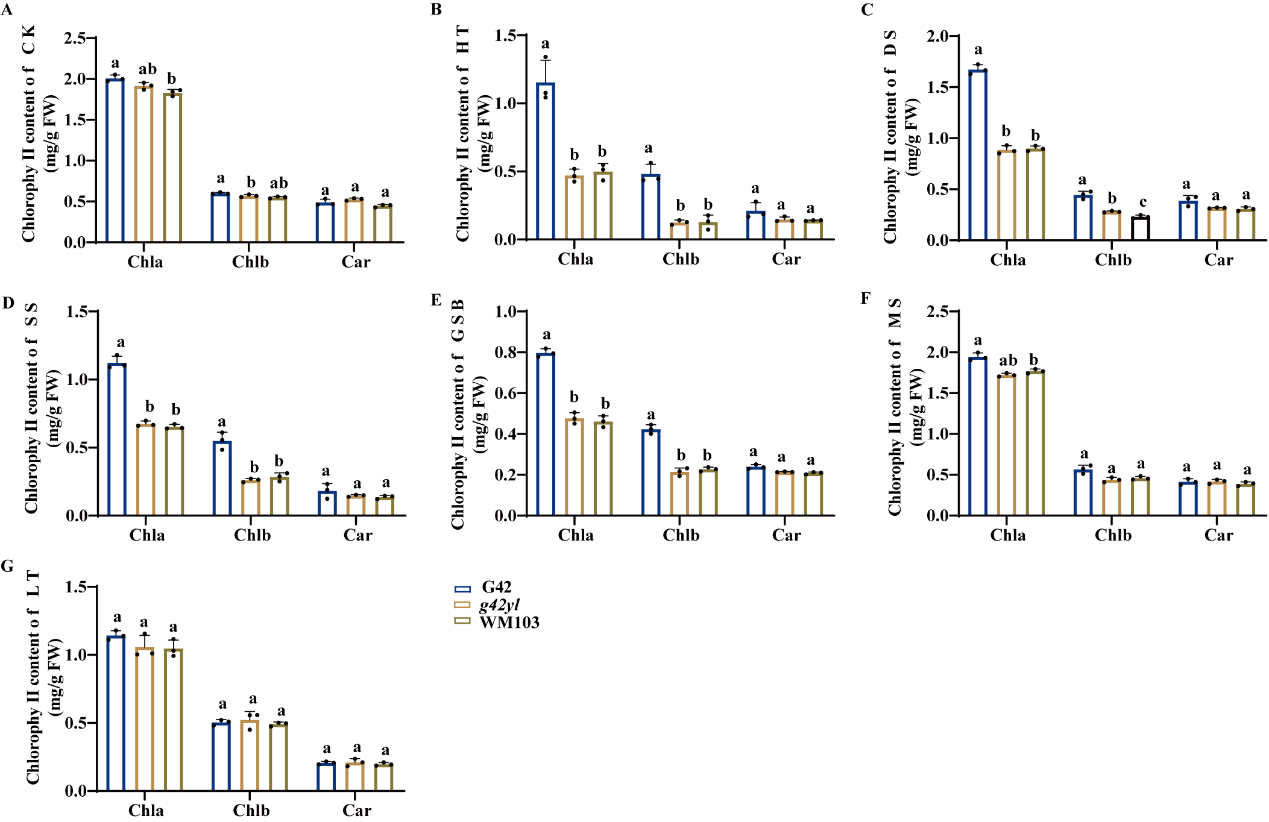


**Fig. S2** **Chla, Chlb, and carotenoid levels in seedlings after 5 days of various stress treatments.** Data: mean ± SD (n=3). Different letters: significant differences (*P* < 0.05, Tukey’s HSD test). CK, control; HT, high temperature (day/night, 45℃/30℃); DS, drought stress; SS, salt stress (250 mM NaCl, bottom irrigation); GBS, gummy stem blight; MS, mechanical stress; LT, low temperature (day/night, 10℃/4℃).


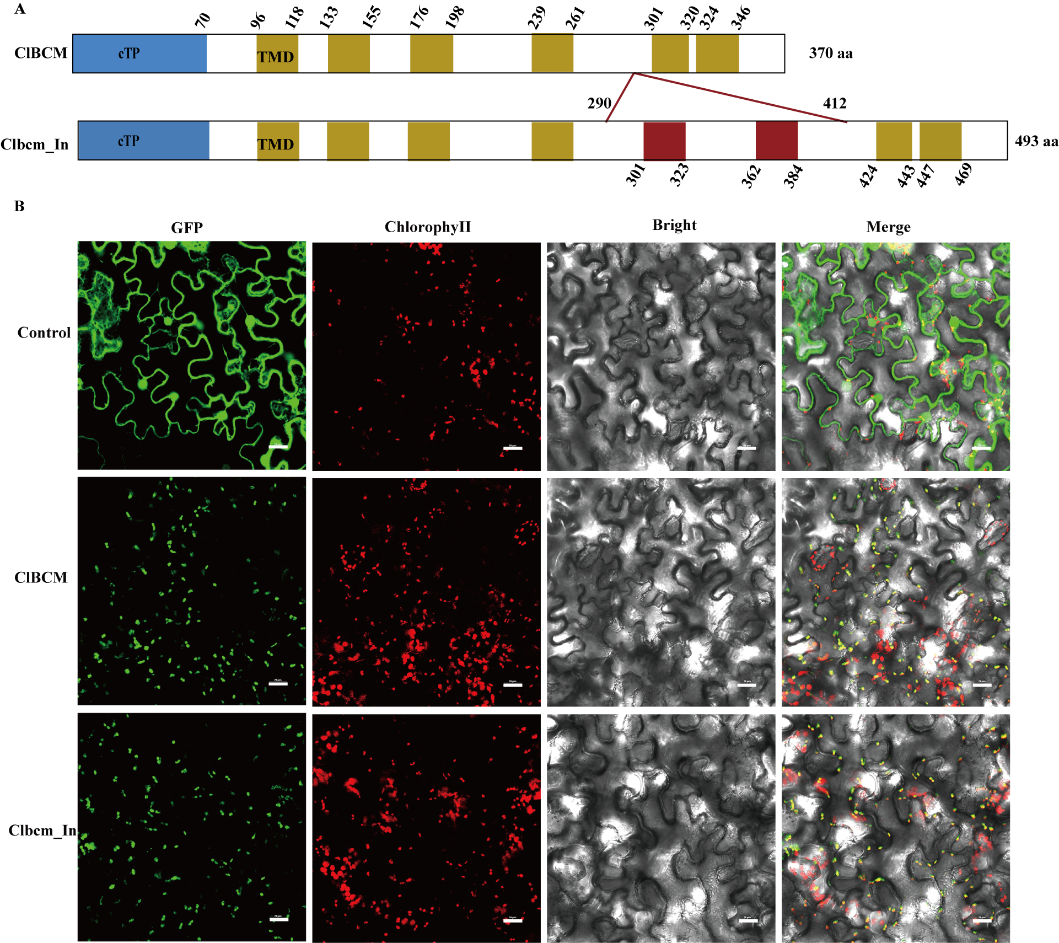


**Fig. S3 The protein structure and subcellular localization of *ClBCM* and *Clbcm_In***. (A) cTP, chloroplast transit peptide; TMD, transmembrane domain; Red-line region: the additional protein sequence in the mutant to the wild-type. (B) Subcellular localization of the *ClBCM*, *Clbcm_In*-GFP fusion protein in tobacco. Scale bars=20 um.


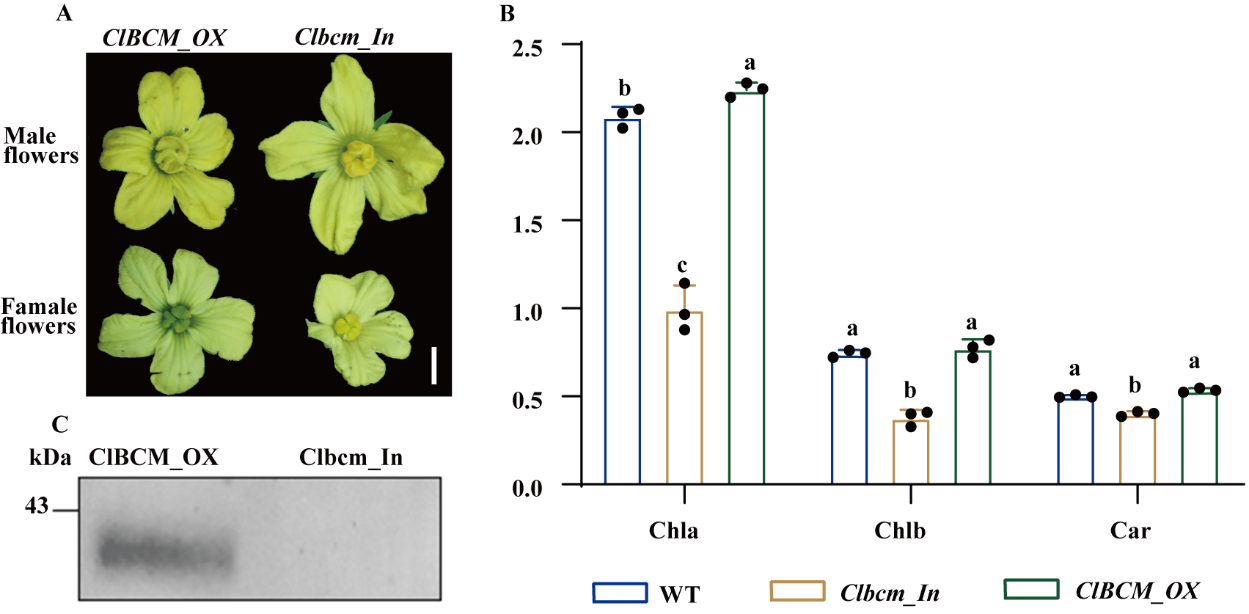


**Fig. S4 Overexpression of *ClBCM* rescues the mutant phenotype.** (A) *ClBCM_OX* and *Clbcm_In* phenotype, scale bar=2cm. (B) Chla, Chlb, and carotenoid levels of adult plants after 5 days of high temperature. Data: mean ± SD (n=3). Different letters: significant differences (*P* < 0.05, Tukey’s HSD test). (C) FLAG-immunoblot analysis of ClBCM_OX protein expression via SDS-PAGE. SDS-PAGE, sodium dodecyl sulphate-polyacrylamide gel electrophoresis.


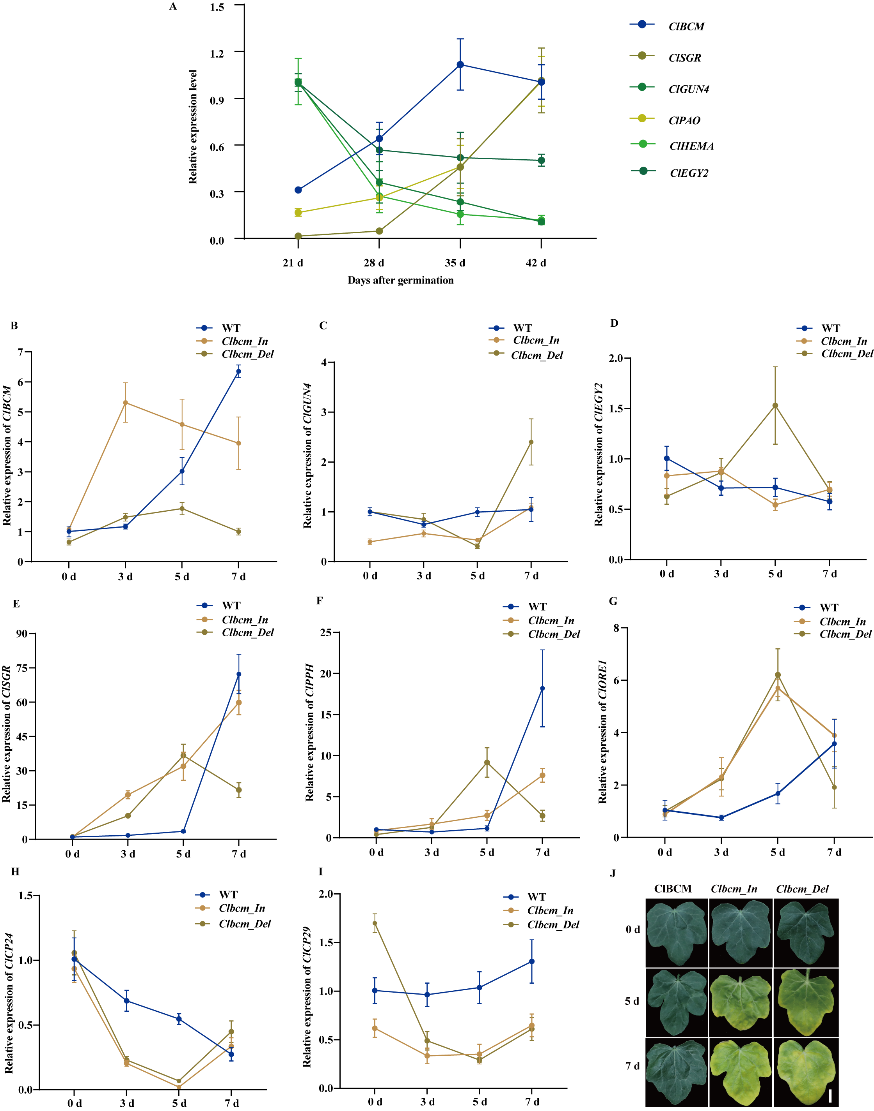


**Fig. S5 Expression of *ClBCM* and related genes in WT and mutant seedlings.** (A) Normal condition (day/night, 25°C/20℃). Gene expression was normalized to 42-day-old WT seedlings for *ClBCM*, *ClSGR*, and *ClPAO*, and to 21-day-old WT seedlings for *ClGUN4*, *ClHEMA*, and *ClEGY2*. Data: mean ± SD (n=4). (B-I) High temperature (HT, day/night, 45°C/30℃). Expression levels are presented relative to that in WT seedlings at 0 d. Data: mean ± SD (n=3). (J) Phenotypes sampled at different periods (days after HT treatment). Scale bar= 1 cm.


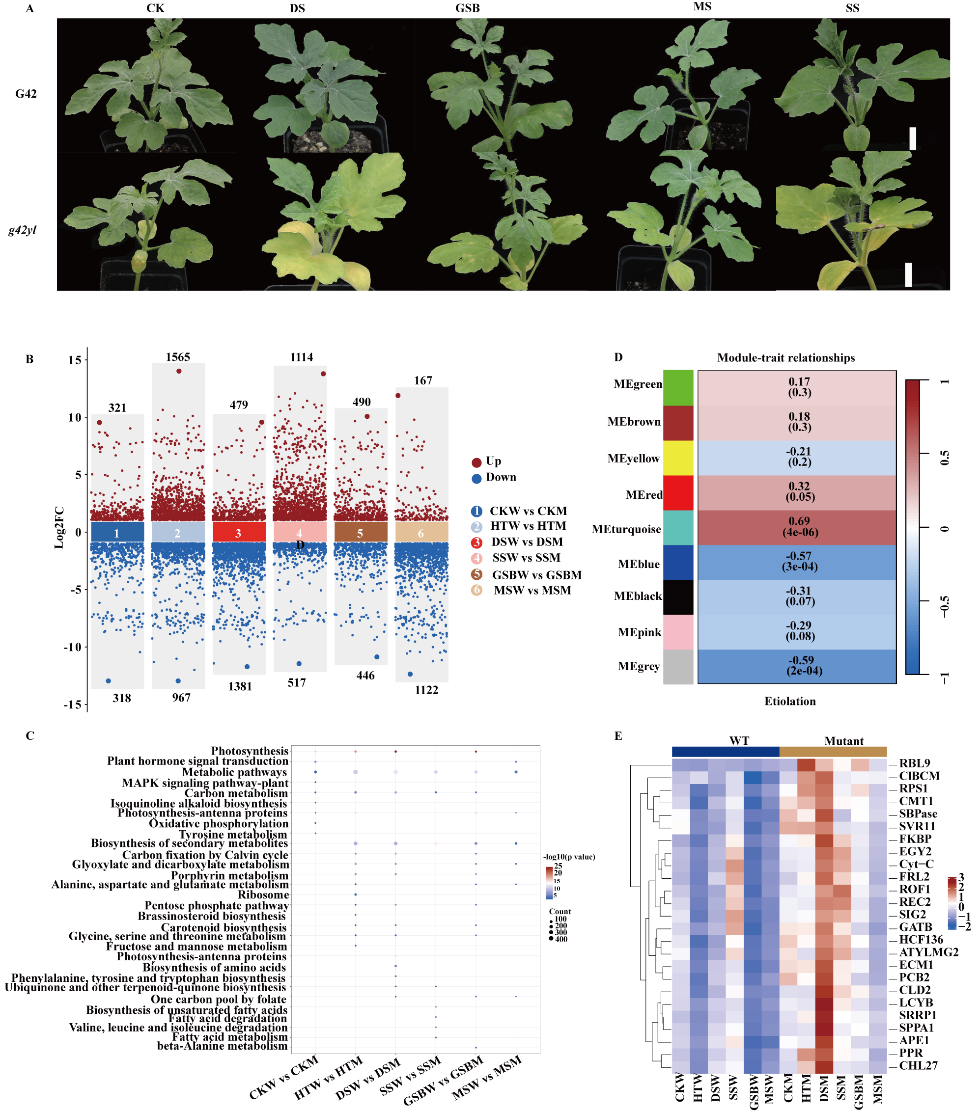


**Fig. S6** **RNA-seq analysis between G42 and *g42yl*.** (A) Phenotypes following 5-Day stress treatment for transcriptome sequencing. CK, control; DS, drought stress; GSB, gummy stem blight; MS, mechanical stress; SS, salt stress (250 mM NaCl, bottom irrigation). (B) Volcanic plots of differentially expressed genes (DEGs). Red and blue points represent the up-regulated and down-regulated DEGs, respectively. CKW and CKM represent the controls of G42 and *g42yl*, respectively. HT, high temperature (day/night, 45℃/30℃). (C) KEGG enrichment analysis for the DEGs. Bubble size: the number of genes; Color: the adjusted *P* value. (D) Identification of leaf yellowing-associated modules via WGCNA. WGCNA, Weighted gene co-expression network analysis. The numbers in the module represent correlation coefficients, while those in parentheses indicate the *P*-value. (E) Expression clustering of *ClBCM* and twenty-three WGCNA-identified genes in WT and mutant under stress. Data: log₂(TPM+1) (Transcripts Per Million) transformed and row-scaled.
